# Medical Ozone: A Redox Regulator with Selectivity for Rheumatoid Arthritis Patients

**DOI:** 10.3390/ph17030391

**Published:** 2024-03-19

**Authors:** Olga Sonia León Fernández, Gabriel Takon Oru, Renate Viebahn-Haensler, Gilberto López Cabreja, Irainis Serrano Espinosa, María Elena Corrales Vázquez

**Affiliations:** 1 Pharmacy and Food Institute, University of Havana, Havana 13603, Cuba; orugabriel@yahoo.com; 2 Medical Society for the Use of Ozone in Prevention and Therapy, D-76473 Iffezheim, Germany; renateviebahn@t-online.de; 3 National Institute of Rheumatology, Ministry of Public Health, Havana 19210, Cuba; gilberto.lopez@infomed.sld.cu (G.L.C.); serranoespinosairainis@gmail.com (I.S.E.); 4 Clinical Laboratory of the Surgical and Clinical Teaching Hospital “10 de Octubre”, Faculty of Medical Sciences, Ministry of Public Health, Havana 19210, Cuba; mariaelenacorralesvazques@gmail.com

**Keywords:** ozone, rheumatoid arthritis, osteoarthritis, oxidative stress

## Abstract

Rheumatoid arthritis (RA) and osteoarthritis (OA) are the most common arthritic diseases. Medical ozone has demonstrated its effectiveness in combination therapy with methotrexate or non-steroidal anti-inflammatory drugs for RA and OA, respectively. Although RA and OA have been compared from different points of view, few studies have considered their redox status in spite of the oxidative processes that are involved in both diseases. The aim of this study was to compare RA with OA, evaluating their redox status and the effects of ozone on their clinical response to combined therapy with ozone. The redox status of 80 patients was determined: antioxidant defenses, injury markers, two subjective variables (pain and disability), and levels of antibodies against cyclic citrullinated peptides were evaluated. Oxidative stress and clinical response to combined therapy with ozone was higher than in the case of RA. After medical ozone treatment, there was an increase in antioxidant defense and a decrease in injury markers as well as pain, disability, and autoantibody concentrations. Redox biomarkers were able to differentiate between both arthritic diseases and combined therapy with ozone (methotrexate + ozone), showing a therapeutic selectivity for RA in comparison with OA.

## 1. Introduction

Rheumatoid arthritis (RA) and osteoarthritis (OA) are the most common forms of arthritis with a global impact of over 300 and almost 20 million people, respectively. In addition, this picture is increased due to life expectancy, in addition to the aging of the worldwide population [1]. RA prevalence has been estimated to be between 0.5 and 1% across the global level in 2002, in which the number of females is double that of males [2]: here, the frequency of OA, the increase in which is very high in the aging and obese population, is predicted to have increased to 67 million by 2030 [3]. Both diseases share common factors in the context of their pathophysiology, although they are of different origins [4,5].

RA is a chronic systemic autoimmune disease causing progressive disability and premature death [6]. It is a symmetric peripheral disease involving bone erosion, proximal involvement, and destructive bone lesions. In addition to this, RA patients display synovitis, morning stiffness, and/or immobility of their proximal interphalangeal joints [7]. Finally, they suffer from joint failure as a result of cartilage damage, and severely weakened tendons and ligaments [8]. On the other hand, OA is the most common degenerative joint disease. Its clinical manifestations include asymmetric peripheral disease, distal and proximal involvement, and synovitis, together with stiffness and/or immobility of the proximal interphalangeal joints, sclerosis, bone spurs, and joint space narrowing [7]. The disease displays an abnormal process of joint tissue remodeling and it is viewed as a disease affecting the whole joint, with mainly the knee and hip [9] contributing the most to the disease burden. Other differences between RA and OA are the pharmacological treatments. RA therapies are primarily based on disease-modifying antirheumatic drugs (DMARDs) such as methotrexate (MTX), which is considered to be the gold standard, chloroquine, and anti-TNF drugs as biological therapy, etc., whereas OA is treated basically with non-steroidal anti-inflammatory drugs and other options [10]. However, despite the use of RA-DMARDs, the clinical signs sometimes persist. Different factors are involved; among them, the existence of variability in the individual’s response to DMARDs [11], so other therapeutic strategies are open and should be developed, among them medical ozone, which could become an alternative treatment for RA and OA.

Medical ozone is an ozone/oxygen mixture administered at low concentrations. It is able to re-establish cellular redox balance and regulate other mediators through an ozone oxidative pre-postconditioning mechanism [12]. The proposed mechanism has been validated in pathological conditions such as ischemic syndrome, diabetes and diabetic foot, disc hernia, pain, and other diseases [13].

The ultimate therapeutic goal in RA treatment is remission, or at least low disease activity, which may not always be achieved with MTX monotherapy, so that a combination therapy appears to be better.

When MTX is combined with medical ozone, the beneficial clinical response of RA patients are increased in comparison with those patients who receive no ozone [14].

RA and OA patients are frequently seen in a rheumatology context and the diseases are compared not only because of their frequency in the field of rheumatology, but also because of the burden of disease that they produce [15]. Only a few studies have contrasted both arthritic diseases: vascular age was not different when RA and OA were compared [10], and leptin is a potential therapeutic target of the degenerative process in rheumatic patients. Nevertheless, concrete mechanisms of action of leptin in RA and OA are still not completely clear [16]; matrix metalloproteinases are synthesized by chondrocytes and synovial fibroblasts, and their expression is elevated in cartilage and synovial tissues in patients suffering from RA and OA without apparent differences [17]. At the same time, exogenous adenosine exerts anti-inflammatory effects in the synoviocytes of osteoarthritis (OA) and rheumatoid arthritis (RA) patients through interactions with adenosine receptors (ARs). These contribute to anti-inflammatory effects [18]. Synoviocytes of both arthritic diseases express the complete enzymatic machinery to synthesize adenosine/inosine; however, differences between RA and OA were found: OA synoviocytes produced significantly higher nucleoside concentrations compared to RA cells. The expression of A_2A_AR (subtype 2A adenosine receptor) and A_2B_AR (subtype 2B adenosine receptor) seemed to be more pronounced in RA synovial tissue and most effects of inhibitors and agonists were stronger in RA than in OA synoviocytes. In the light of these factors, the aim of this study was to investigate whether medical ozone displays any therapeutic selectivity for a particular arthropathy as well as whether the patients’ response to ozone therapy may enable us to identify the differences between RA and OA diseases from a redox point of view.

## 2. Results

Baseline patients characteristics are shown in Table 1. Women were the predominant sex in RA groups. As stated above, the number of females concerned is double that of males [2], although an explanation for this phenomenon has not yet been found. A similar picture in the OA groups can also be observed. No differences between the ages of RA and OA patients were found. The OA grading in patients receiving the combined therapy MTX + ozone is noteworthy, since the severity of knee lesions was higher with regard to patients who received NSAIDs.

Disease development time was longer in RA compared to the OA groups, although no differences were observed among RA Control (MTX) vs. Ozone-treated RA (MTX + ozone) and OA Control (NSAIDs) vs. Ozone-treated OA (NSAIDs + ozone) group. Ethnic origin showed opposite pictures between RA and OA: Caucasians were predominant in OA whereas non-Caucasian was the prevailing ethnic origin in the case of RA.

### 2.1. Antioxidant Defenses and Injury Biomarkers in RA and OA Patients

An analysis of redox status in both diseases evidenced that RA oxidative stress was higher compared with OA patients; this could be classified as severe and moderate for RA and OA, respectively.

**Figure 1:**
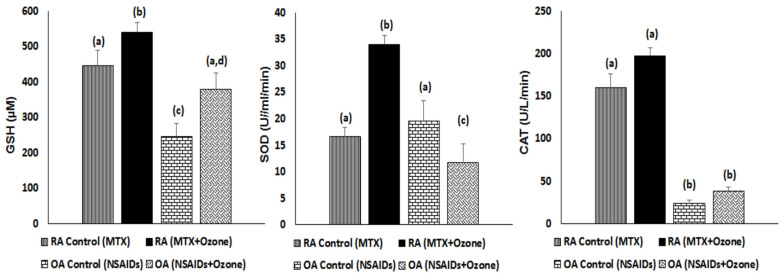
Antioxidant defenses in RA and OA patients after the combined therapies MTX + ozone and NSAIDs + medical ozone, respectively. The data are represented as mean ± S.E.M. Mean values with different letters indicate significant differences (*p* < 0.05) between the diseases: RA, before (MTX) vs. after (MTX + Ozone) and OA, before (NSAIDs) vs. after (NSAIDs + Ozone). GSH, reduced glutathione; SOD, superoxide dismutase activity; CAT, catalase activity. Statistical tests: one-way analysis of variance (ANOVA) followed by Student Newman–Keuls tests.

RA antioxidant defenses increased after MTX + Ozone treatment, whereas OA showed a different picture. Reduced glutathione (GSH) displayed a significant increment similar to that with OA, although total GSH levels in OA were less than in RA patients. After combined therapy, the activity of SOD—a scavenger of superoxide radicals—showed a similar course to GSH, with a significant decrease in the OA group. Although catalase activity was higher in RA than in OA patients, there were no differences in either disease following pharmacological therapy (Figure 1).

Injury redox biomarkers (OPT and MDA) were higher in the case of RA compared with OA, similar to the picture for antioxidant defenses and the intensity level of oxidative stress.

**Figure 2:**
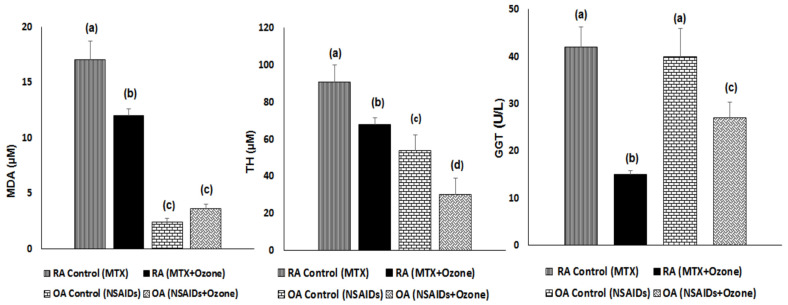
Injury biomarkers and GGT activity in RA and OA patients after the combined therapies MTX + ozone and NSAIDs + medical ozone, respectively. The data are represented as mean ± S.E.M. Mean values with different letters indicate significant differences (*p* < 0.05) between the diseases: RA, before (MTX) vs. after (MTX + Ozone) and OA, before (NSAIDs) vs. after (NSAIDs + Ozone). TH, total hydroperoxides, MDA, malondialdehyde; GGT, gamma-glutamyl transferase. Statistical tests: one-way analysis of variance (ANOVA) followed by Student Newman–Keuls test.

After combined therapy with medical ozone, a decrease in TH levels was observed in both diseases, which were high prior to pharmacological treatment. TH is an indicator of hydrogen peroxide produced by SOD activity and a precursor of hydroxyl radicals that trigger lipid peroxidation with MDA generation. The fact that MDA contributes to the generation of new epitopes able to produce autoantibodies in RA has already been described [19]. MDA decreased in RA after medical ozone, but not in the OA group (Figure 2).

Increased gamma-glutamyl transferase (GGT) activity has been linked with loss of antioxidant–pro-oxidant balance as well as a producer of oxidative stress. The cleavage of GSH (low molecular weight antioxidant) is the most important physiological function of GGT [20]. In addition, GGT elevations along with GSH depletions have been associated with different diseases having a high mortality rate, even including RA [21]. Both the RA and the OA groups showed a decrease in GGT activity after ozone treatment, more markedly in RA patients (Figure 2), which was in line with the increase and decrease in antioxidant defenses and injury biomarkers, respectively.

### 2.2. Pain, Disability, and Anti-Cylic Citrullinate Peptide Levels in RA and OA Patients

Pain and disability are very close parameters (Table 2). OA patients displayed higher pain intensity than patients with RA. After combined therapy with medical ozone, there was a decrease in pain perception in both diseases; this was more marked in the case of RA, which showed statistical differences regarding all groups. These results were in line with disability and anti-CCP levels. In RA patients, medical ozone promoted a decrease with statistically significant differences, whereas in the case of OA there was only a tendency towards a slight decrease.

**Figure 3:**
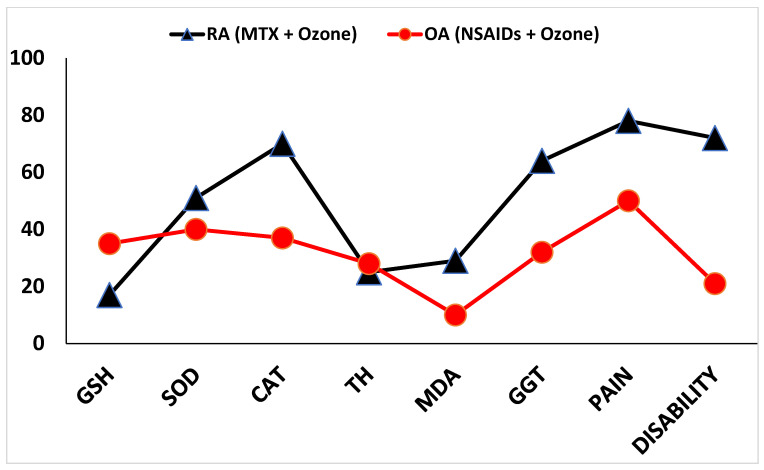
Ozone induced improvement on each biomarker in RA and OA patients (in %). GSH, reduced glutathione; SOD, superoxide dismutase activity; CAT, catalase activity; TH, Total Hydroperoxides, MDA, malondialdehyde; GGT, ganma glutamyl transferase.

Combined therapy with medical ozone led to an improvement in each redox biomarker, together with pain and disability (Figure 3).

In general, both diseases displayed an increase in beneficial clinical response following medical ozone treatment. This was higher in RA patients treated with MTX + ozone, with a 75% improvement in markers (SOD, CAT, MDA, GGT, pain and disability) compared to OA patients.

**Figure 4:**
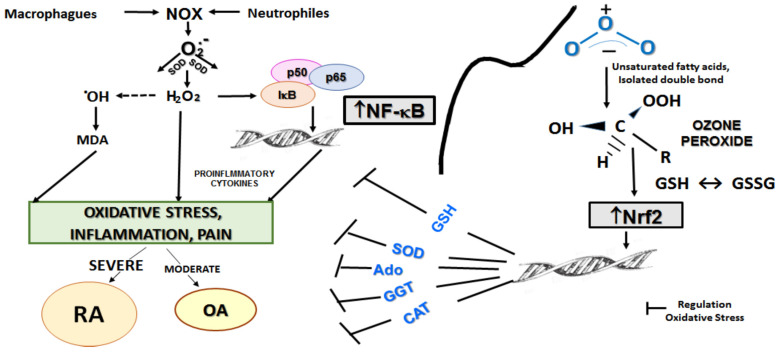
The role of medical ozone in antioxidant/pro-oxidant balance in arthritic diseases. The overproduction of superoxide radicals caused by NADPH oxidase (Nox) results in these being metabolized to hydrogen peroxide: this, along with superoxide radicals is able to generate hydroxyl radicals and lipid peroxidation (MDA). In addition to this, nuclear factor kappa B (NF-kB) is activated by ROS, involving pro-inflammatory cytokine production. The impact of oxidative stress, inflammation, and pain in RA and OA are correspondingly severe or moderate, respectively. While the generation of ozone peroxides activates the nuclear factor Nrf2, erythroid 2-related factor 2: this in turn produces an antioxidant network that regulates the oxidative imbalance through different molecules and mechanisms. RA, rheumatoid arthritis: OA, osteoarthritis; Nox, NADPH oxidase; GSH, reduced glutathione; SOD, superoxide dismutase activity; Ado, adenosine; GGT, γ glutamyl transferase; CAT, catalase activity.

Figure 4 shows different pathways associated with cartilage damage and bone destruction in RA and OA, as well as ozone’s protective mechanisms due to the participation of different molecules activated by ozone peroxides. The regulation of oxidative stress seems to be critical for the progression of RA and OA, which allows us to differentiate between these arthritic diseases by the degree of loss of cellular redox balance.

## 3. Discussion

RA and OA are the two most common diseases in rheumatology consultations. The results of this study have shown that they differ from each other in the intensity of oxidative stress, as well as in RA’s selectivity of medical ozone in the therapeutic response to treatment—employed as combined therapy MTX + medical ozone—compared with OA.

The generation of ROS together with a decrease in antioxidant defenses generates oxidative stress of different intensity in RA and OA. This promotes inflammation of the joints, ligaments, tendons, bones and/or muscles, and even, in some cases, damage to other organs [22]. Inflammation and oxidative stress are influenced by two transcription factors: NF-kB (nuclear transcription factor kappa B) and Nrf2 (nuclear factor erythroid 2-related factor 2), respectively. Both exert opposite functions in the inflammatory process and have been considered to be the “Yin and Yang” of inflammatory response [23]. These transcription factors are regulated by medical ozone [24,25]; hence the different genes involved in the effectiveness of ozone and the targets associated with its therapeutic effects.

The combined therapy of MTX + medical ozone not only improved the patient’s redox status, but also demonstrated therapeutic selectivity for RA compared to OA with the redox parameters. This specificity was expressed in an improvement in the antioxidant/pro-oxidant balance of the patients (Figure 3). Medical ozone improved 75% of the evaluated biomarkers (with GGT and CAT being the outstanding variables), including pain and disability that represent patient assessments of their physical state in carrying out everyday activities. Despite treatment with MTX—the first-line DMARDs for this autoimmune disease—patients showed severe oxidative stress, and the beneficial effects of ozone were greater for RA compared to OA. This result suggests that the therapeutic targets regulated by ozone in RA involve other signaling mechanisms or receptors that are not as prominent in OA.

The significant increase in pain in OA is worthy of note in the context of all treatments. These results were consistent with those reported in a comparative study between RA and OA where vascular aging, cardiovascular risk, and inflammation were evaluated. In this study, in addition to pain, patients with OA showed fatigue, disability, and a poor quality of life, compared to patients with RA [10] (Figure 3, Table 2).

The beneficial effects of the introduction of medical ozone in conventional therapy demonstrated its effectiveness for the treatment of these arthropathies. In line with these results, previous studies showed that ozone “per se” was capable of reducing inflammation and regulating oxidative stress and TNF-α and IL-1β mRNAs in a chronic model of RA [26].

Medical ozone is a pleiotropic substance regulating different therapeutic targets (Figure 4). Glutathione depletion has been identified as the main redox component of autoimmune diseases [27]. The significant increase in GSH in RA and OA is one of the results that explains the regulatory and beneficial effects of medical ozone on the redox status of patients. The oxidation of GSH, or cysteine residues, represents a signal in which Nrf2 is activated [28] by ozone peroxides. The activation of this factor promotes a “de novo” synthesis of GSH and other antioxidants; this reduces the availability of hydrogen peroxide, which activates NF-kB and the generation of pro-inflammatory cytokines. In addition, GSH is a cofactor of hydrogen peroxide detoxifying enzymes (GSH peroxidase along with free radicals such as hydroxyl radical).

Simultaneously, the significant decrease in GGT (γ glutamyl transferase) contributes to an increase in the availability of GSH. This effect was more marked in RA than in OA. GGT cleaves the γ glutamyl peptide bond to generate cysteinyl glycine, which is cleaved by non-specific dipeptidases, releasing cysteine and glycine. In this way, GSH is depleted and its antioxidant and other functions disappear. GGT is regulated by medical ozone in patients with RA and OA, and is a marker of the patient’s response to treatment [29,30]; it is activated by oxidants and is associated with Nrf2-mediated signaling mechanisms [31]. It has been considered as a marker of oxidative stress as well as a risk indicator for diseases with high morbidity and mortality (pancreatic cancer [32], stroke [33], osteoporotic fractures [34], etc.).

SOD and CAT are two metabolically related antioxidant enzymes involved in the metabolism of hydrogen peroxide and other ROS (Reactive Oxygen Species). An excess in the production of superoxide anion radicals (NADPH Oxidase, Xanthine Oxidase and others) increases the activity of SOD that transforms the superoxide radical into hydrogen peroxide (TH). This is significantly decreased after combined therapy with medical ozone, with an increase in CAT that reduces this peroxide to water and oxygen. This effect not only decreases the activation of NF-kB but also influences the production of the hydroxyl radical. The superoxide anion not biotransformed by SOD and hydrogen peroxide represents the greatest contribution to ROS [35]. These ROS, in the presence of ferrous iron, generate the hydroxyl radical that oxidizes membrane lipids generating MDA, which in this study decreased significantly after ozone treatment. MDA is one of the recognized markers of oxidative damage in RA [36]. MDA concentrations are high in RA and, coinciding with our results, its levels are higher than in OA. [37]. It has been reported that MDA–acetaldehyde adducts colocalize with citrullinated proteins in patients with RA. On the other hand, anti-MDA–acetaldehyde antibodies strongly correlated with Anti-Cyclic Citrullinate Peptide seropositive patients with RA, suggesting their participation in the disease and its progression [19].

Adenosine is a nucleoside produced by nucleotides, including ATP, and is involved in purinergic signaling mechanisms and anti-inflammatory effects in RA and OA, through “G” protein-coupled receptors. There are four subtypes of these receptors (A1, A2a, A2b and A3), all detected in the synovial tissue of patients with RA and OA. Furthermore, the synoviocytes in both arthritic diseases all have the enzymatic machinery to produce adenosine and inosine, although it is adenosine that fundamentally exerts the biological effects.

Adenosine is another mediator regulated by medical ozone [38,39], which contributes to the antioxidant effects observed in patients with RA and OA. Adenosine has been associated with both antioxidant and anti-inflammatory/pro-inflammatory actions, depending on the expression levels and receptor subtypes involved [18]. In hepatic ischemia/reperfusion processes, ozone preserved adenosine levels and maintained, at the level of the control group, the activity of the enzyme adenosine deaminase, which biotransforms the nucleoside into inosine. The protective effects of medical ozone on liver damage were mediated by the activation of adenosine A1 receptors, one of the most abundant and high-affinity receptors, present in synoviocytes from patients with RA and OA [40]. By using the specific A1 receptor antagonist 2-chloro-N6-cyclopentyladenosine (DPCPX) and the agonist 8 cyclopentyl-1,3-dipropylxanthine (CCPA), it has been demonstrated that the pharmacological effects of medical ozone are mediated by adenosine receptor type A1 [31].

Mitochondria are one of the main sources of ROS. Medical ozone maintains the integrity of mitochondrial structures, evidenced by electronic microscopy studies and reduced oxidative stress [31]. Confirmation that A1 adenosine receptors mediates medical ozone effects was found in carrageenan-induced synovitis and PTZ (pentylenetetrazol)-induced generalized seizures models [41,42]. The increase in the availability of adenosine and the activation of A1 receptors helps to explain not only the redox state but also the improvement of pain and disability. Adenosine A1 receptors have been involved in anti-nociceptive effects and they have been suggested as important peripheral targets for analgesic drug development [43,44]. In line with these results is the decrease in antibodies against citrullinated cyclic peptides, indicating protection against damage to cartilage and erosion of the bone (Table 2).

## 4. Material and Methods

### 4.1. Study Design

This prospective, longitudinal, and randomized study was approved by the joint institutional review board (Scientific and Ethics Committees from the National Institute of Rheumatology, Ministry of Public Health, Cuba, and Pharmacy and Food Institute, University of Havana, Cuba) in accordance with the principles of the Declaration of Helsinki [45]. All patients gave their informed consent to enrollment after receiving adequate information concerning the study (characteristics of the study, benefits and possible side effects). Before enrollment, all participants attended a training program to familiarize them with the study objectives and treatment plans. Eligible patients were randomized using a computer-generated list of random numbers (Research Randomizer Form v 4.0). The random sequence was created using freely accessible tools which uses the pseudo-random number generator developed by [46] and modified by [47]. The demographic characteristics and the medical history of the patients were recorded, and laboratory tests performed.

RA patients received MTX 12.5 mg, intramuscular (i.m.), once/week (every Monday from 9:00–10:00 in the morning) + Ibuprophen (400 mg, oral), one tablet each 8 h + folic acid (5 mg, oral), one tablet/day from Wednesday to Saturday according to the established treatment protocols + medical ozone, whereas OA patients were treated with non-steroidal anti-inflammatory drugs (NSAIDs) and other analgesics + medical ozone. Controls for each arthritic disease were included.

The patients (*n* = 80) were randomized into four different treatment groups (20 each): Group I, Control RA (MTX); Group II, Ozone-treated RA (MTX + Ozone); Group III, Control OA (NSAIDs); Group IV, Ozone-treated OA (NSAIDs + Ozone).

Ozone was generated using an OZOMED unit, Cuba. Patients received 20 treatments via rectal insufflation (five applications per week from Monday through Friday). Concentrations of ozone: 20 mg/L to 35 mg/L were in stepped application as follows: 1st week: 20 mg/L, 100 mL; 2nd week: 25 mg/L, 150 mL; 3rd week: 30 mg/L, 200 mL; and 4th week: 35 mg/L, 200 mL.

All reagents and drugs used in this study came from Sigma Aldrich (St. Louis, MI, USA). When other reagents or procedures were used it has been declared in the article.

### 4.2. Inclusion and Exclusion Criteria

#### 4.2.1. For Rheumatoid Arthritis

Adult patients (40–70 years old) of both sexes and different ethnic origins with a diagnosis of RA who fulfilled the American College of Rheumatology [48] classification criteria for RA were eligible to participate in the study. Patients of the National Institute of Rheumatology, Ministry of Public Health, Cuba, who met the following criteria were chosen: Disease Activity Score 28 (DAS-28) > 3.2 y ≤ 5.1) in patients whose examination was carried out under blinded conditions by a physician different to the one who selected the patients according to a randomized scheme of treatment and a preliminary brief medical history; The Health Assessment Questionnaire-Disability Index (HAQ-DI (+ > 1.25), according to the validated Spanish version) [49]; and anti-Cyclic Citrullinate Peptides ELISA (DRG Instruments GmbH, Marburg, Germany; DRG International, Inc. Springfield, NJ, USA) (anti-CCP > 10 U/mL in serum); in addition, patients with disease duration longer than five years were included.

Exclusion criteria were: (a) patients with any history of chronic conditions such as liver disease, diabetes mellitus, respiratory disorders, cardiovascular diseases, alcohol usage, and smoking; (b) patients with an overlapping syndrome, cancer, or other associated autoimmune disorders, or who were pregnant; and (c) patients who had been receiving corticosteroid agents and were under treatment with disease-modifying antirheumatic drugs different to MTX, anti-TNF or other biological agents, and antioxidants within less than three months before the study date.

#### 4.2.2. For Osteoarthritis

Inclusion criteria were: men and women, aged 40–75, with body mass index (BMI) < 35 kg/m^2^, and OA grades III-IV as an indication for knee arthroscopic resulting from radiographic and arthroscopic classification. Radiographs of both knees were obtained using anteroposterior projection with support, lateral with 30° flexion, and Merchant (45°) views. These patients were waiting for surgery.

Exclusion criteria were: (a) infectious conditions, history of trauma (dislocation or fracture), inflammatory arthritis, microcrystalline arthropathies, history of septic arthritis, ligament injury, non-specific synovitis, angular deformity > 10°, chondral lesions G-IV, outer bridge (>1 cm^2^), neoplasms, or allergy to any of the components of the products under study; and (b) patients taking antioxidant agents within less than three months.

#### 4.2.3. Evaluation of Activity in Both Diseases

Changes in the evolution of RA, through suitable indexes of activity (clinical parameters: DAS-28, HAQ-DI (disability), Visual Analog Scale 10–100, “10” (minimum pain intensity) and “100” (maximum pain intensity). No pain was considered as “0”. Anti-CCP antibodies and redox status determinations before the beginning and at the end of clinical study (21 days) were assessed. Each patient was his/her own control (i.e., before medical ozone treatment). With regard to OA, pain, redox status, and disability of patients through Lysholm Knee Scoring Scale (Virginia Therapy and Fitness Center) were evaluated.

### 4.3. Biochemical Determinations

Blood samples for biochemical analysis were obtained after a 12 h overnight fast, at the beginning, and 24 h after the last ozone treatments in both diseases.

Anti-CCP antibody in serum was determined by ELISA (DRG, DRG Diagnostics, GmbH, Germany) (sensitivity 90%, specificity 98.3%, and diagnostic efficacy 95.3%). Erythrocyte sedimentation rate (ESR) was determined using the quantitative method of Westergren [50].

Redox parameters (serum) were determined by spectrophotometric methods using a reader plate Ultra Microanalytic System (Immunoassay from Cuba) and BOECO Model S220 Spectrophotometer, from Germany. Superoxide dismutase (SOD) activity was measured using kits supplied by Randox Laboratories Ltd., Ireland, UK (Cat. No. SD125 and No. RS505). Catalase (CAT) activity was determined by following the decomposition of hydrogen peroxide at 240 nm at 10 s intervals for 1 min [51]. After precipitation of thiol proteins using trichloroacetic acid 10%, reduced glutathione (GSH) was measured according to the method of [52] with Ellman’s reagent [5′5 dithiobis (2-nitrobenzoic acid) 10^−2^ M (Sigma St. Louis, MO, USA)]; absorbance was measured at 412 nm. Quantification of total hydroperoxides (TH) was accomplished with a Bioxytech H_2_O_2_-560 kit (Oxis International Inc., Portland, OR, USA). Concentrations of Malondialdehyde (MDA) were analyzed using the LPO-586 kit obtained from Calbiochem (La Jolla, CA, USA).

### 4.4. Statistical Analysis

The OUTLIERS preliminary test for detection of error values was applied. Following this, data were analyzed via one-way analysis of variance (ANOVA) followed by a homogeneity variance test (Bartlett–Box). In addition, a multiple comparison test was used (Student Newman–Keuls test). The Student *t*-test for independent samples, canonical discriminant analysis, and *t*-tests of paired samples were applied. Results are presented as means ± standard error of the mean (S.E.M.). The level of statistical significance used was at least *p* < 0.05.

## 5. Conclusions

RA and OA showed significant differences in the intensity of their oxidative stress associated with the pathogenesis of the disease: this was expressed in quality-of-life indicators such as pain and disability. Medical ozone, in the form of combined therapy with MTX or NSAIDs, improved the antioxidant/pro-oxidant balance of the patients. However, the beneficial effects of medical ozone showed selectivity for RA in the generality of the redox biomarkers studied. Based on these results, another study is being developed with the aim of increasing the population sample and studying other subtypes of receptors involved in the observed effects, as well as medical ozone effectiveness in early RA.

The strengths of this research work have been the determination of the redox status in RA and OA patients, as documented in our previous extensive experimental studies in animals which demonstrated the efficacy and security of systemic medical ozone and its mechanism of action, which has greatly contributed to the increase in the use of MTX + medical ozone as combined therapy in different Rheumatology Services in Cuba. The main limitation of this work has been the sample size (80 patients), so at present there is another study in progress with the aim of increasing the population sample.

## Figures and Tables

**Table 1:** Clinical picture of RA and OA patients.

Demographic Data/Patient Histories		Rheumatoid Arthritis (RA)		Osteoarthritis (OA)	
	Groups	Control (MTX) (*n* = 20)	MTX + Ozone (*n* = 20)	Control (NSAIDs) (*n* = 20)	NSAIDs + Ozone (*n* = 20)
Women (*n*/%)		13/65	14/70	15/75	17/88
Men (*n*/%)		7/35	6/30	5/25	3/12
Age (years)		53 ± 8 ^(a)^	57 ± 7 ^(a)^	60 ± 8 ^(a)^	57 ± 9 ^(a)^
Osteoarthritis grading ^(^*^)^					
III		-	-	46%	27%
III–IV		-	-	54%	63%
IV		-	-	-	10%
Previous therapy					
Methotrexate (*n*/%)		20/100	20/100	-	-
NSAIDs		20/100	20/100	20/100	20/100
Folic acid		20/100	20/100	-	-
Evolution time of the					
disease (years)		7 ± 2 ^(a)^	11 ± 3 ^(a)^	4 ± 2 ^(a)^	4 ± 9 ^(a)^
Ethnic origin					
Caucasian		7/35%	9/45%	15/75%	14/70%
Non-Caucasian		13/65%	11/55%	5/25%	6/30%

(*) Assessed by Kellgren and Lawrence Scale, NSAIDs, non-steroidal anti-inflammatory drugs; MTX (Control group): methotrexate + ibuprofen + folic acid; MTX + ozone group: same MTX (Control group) + medical ozone; OA (Control group): OA + NSAIDs; OA + ozone group: same OA (Control group) + medical ozone. The data reflecting age and progress through time of the disease are: mean ± S.E.M of each group. Mean values with different letters indicate significant differences (*p* < 0.05) between both groups MTX vs. MTX+Ozone and NSAIDs vs. NSAIDs + Ozone. Statistical tests: OUTLIERS preliminary test and one-way analysis of variance (ANOVA) followed by Student Newman–Keuls test.

**Table 2:** Medical ozone effects on anti-CCP levels and disability in arthritic diseases.

Groups		Control (MTX)	MTX + Ozone	Control (NSAIDs)	NSAIDs + Ozone
	Markers				
Pain		5 ± 0.2 ^(a)^	1 ± 0.8 ^(b)^	6.6 ± 0.4 ^(c)^	3.3 ± 0.2 ^(d)^
HAQ-DI ^(^*^)^		0.6 ± 0.1 ^(a)^	0.16 ± 0.05 ^(b)^	-	-
Lysholm Scale ^(^**^)^		-	-	64 ± 9 ^(e)^	79 ± 9 ^(f)^
Anti-CCP ^(^***^)^		110 ± 9 ^(a)^	89 ± 8 ^(b)^	.	-

(*) Health Assessment Questionnaire Disease Index in rheumatoid arthritis. “0” indicates the best health status. (**) Lysholm knee scoring scale is a questionnaire designed in order to know how the patient’s knee problems have affected her/his ability to manage in everyday life. Maximal value is 100 points and indicates the best knee status. (***) Anti-Cyclic Citrullinate Peptides (U/L). The data are presented as mean ± S.E.M. Mean values with different letters indicate significant differences (*p* < 0.05) between the diseases: RA, (a) before (MTX) vs. (b) after (MTX + Ozone) and OA, (c,e) before (NSAIDs) vs. (d,f) after (NSAIDs + Ozone). Statistical tests: OUTLIERS preliminary test and one-way analysis of variance (ANOVA) followed by Student Newman–Keuls test.

## Data Availability

Data is unavailable due to ethical and professional restrictions. There is another investigation in progress very close related with the results of this article.

## Abbreviations

ARs	Adenosine receptors
A_2A_R	Subtype 2A adenosine receptor
A_2B_R	Subtype 2B adenosine receptor
A_3_R	A_3_ Adenosine receptor
Ado	Adenosine
Anti-CCP	Anti-Cylic Citrullinate Peptides
CCPA	8 cyclopentyl-1,3-dipropylxanthine
CAT	Catalase activity
ELISA	Enzyme-linked immunosorbent assay
DAS-28	Disease Activity Score 28
DMARDs	Disease-modifying antirheumatic drugs
DPCPX	2-chloro-N6-cyclopentyladenosine
GSH	Reduced glutathione
GGT	γ glutamyl transferase
GSSG	Oxidized glutathione
HAQ-Di	Health Assessment Questionnaire-Disability Index
H_2_O_2_	Hydrogen peroxide
MDA	Malondialdehyde
MTX	Methotrexate
Nox	NADPH oxidase subunit
NSAIDs	Non-steroidal anti-inflammatory drugs
NF-κB	Nuclear factor kappa B
Nrf2	Erythroid 2-related factor 2
OA	Osteoarthritis
·OH	Hydroxyl radical
PTZ	Pentylenetetrazole
RA	Rheumatoid arthritis
ROS	Reactive Oxygen Species
SOD	Superoxide dismutase activity
TH	Total Hydroperoxide

## References

[1] GBD 2017 Disease and Injury Incidence and Prevalence Collaborators (2018). Global, regional, and national incidence, prevalence, and years lived with disability for 354 diseases and injuries for 195 countries and territories, 1990–2017: A systematic analysis for the global burden of disease study 2017. Lancet.

[2] Myasoedova E., Crowson C.S., Turesson C., Gabriel S.E., Matteson E.L. (2011). Incidence of extraarticular rheumatoid arthritis in Olmsted County, Minnesota, in 1995–2007 versus 1985–1994: A population-based study. J. Rheumatol..

[3] Hootman J.M., Helmick C.G. (2006). Projections of US prevalence of arthritis and associated activity limitations. Arthritis Rheum..

[4] Kang L., Dai C., Wang L., Pan X. (2022). Potential biomarkers that discriminate rheumatoid arthritis and osteoarthritis based on the analysis and validation of datasets. BMC Musculoskelet. Disord..

[5] Kim G.M., Park H., Lee S.Y. (2022). Roles of osteoclast-associated receptor in rheumatoid arthritis and osteoarthritis. Jt. Bone Spine.

[6] Guo Q., Wang Y., Xu D., Nossent J., Pavlos N.J., Xu J. (2018). Rheumatoid arthritis: Pathological mechanisms and modern pharmacologic therapies. Bone Res..

[7] Saalfeld W., Mixon A.M., Zelie J., Lydon E.J. (2021). Differentiating Psoriatic Arthritis from Osteoarthritis and Rheumatoid Arthritis: A Narrative Review and Guide for Advanced Practice Providers. Rheumatol. Ther..

[8] Bullock J., Rizvi S.A., Saleh A.M., Ahmed S.S., Do D.P., Ansari R.A., Ahmed J. (2018). Rheumatoid arthritis: A brief overview of the treatment. Med. Princ. Pract..

[9] Katz J.N., Arant K.R., Loeser R.F. (2021). Diagnosis and treatment of hip and knee osteoarthritis: A review. JAMA.

[10] Rodríguez-Vargas G.-S., Santos-Moreno P., Rubio-Rubio J.-A., Bautista-Niño P.-K., Echeverri D., Gutiérrez-Castañeda L.-D., Sierra-Matamoros F., Navarrete S., Aparicio A., Saenz L. (2022). Vascular Age, Metabolic Panel, Cardiovascular Risk and Inflammaging in Patients with Rheumatoid Arthritis Compared with Patients with Osteoarthritis. Front. Cardiovasc. Med..

[11] Conti V., Corbi G., Costantino M., De Bellis E., Manzo V., Sellitto C., Stefanelli B., Colucci F., Filippelli A. (2020). Biomarkers to Personalize the Treatment of Rheumatoid Arthritis: Focus on Autoantibodies and Pharmacogenetics. Biomolecules.

[12] León O.S., Menéndez S., Merino N., Castillo R., Sam S., Pérez L., Cruz E., Bocci V. (1998). Ozone oxidative preconditioning: A protection against cellular damage by free radicals. Mediat. Inflamm..

[13] León O.S. (2014). Ozone Therapy. Oxidative Condition. Basis for Its Clinical Effectiveness.

[14] Fernández O.S.L., Viebahn-Haensler R., Cabreja G.L., Espinosa I.S., Matos Y.H., Roche L.D., Santos B.T., Oru G.T., Vega J.C.P. (2016). Medical ozone increases methotrexate clinical response and improves cellular redox balance in patients with rheumatoid arthritis. Eur. J. Pharmacol..

[15] Ackerman I.N., Pratt C., Gorelik A., Liew D. (2018). Projected Burden of Osteoarthritis and Rheumatoid Arthritis in Australia: A Population-Level Analysis. Arthritis Care Res..

[16] Eldjoudi D.A., Barreal A.C., Gonzalez-Rodríguez M., Ruiz-Fernández C., Farrag Y., Farrag M., Lago F., Capuozzo M., Gonzalez-Gay M.A., Varela A.M. (2022). Leptin in Osteoarthritis and Rheumatoid Arthritis: Player or Bystander?. Int. J. Mol. Sci..

[17] Pulik Ł., Łęgosz P., Motyl G. (2023). Matrix metalloproteinases in rheumatoid arthritis and osteoarthritis: A state of the art review. Rheumatology.

[18] Sohn R., Junker M., Meurer A., Zaucke F., Straub R.H., Jenei-Lanzl Z. (2021). Anti-Inflammatory Effects of Endogenously Released Adenosine in Synovial Cells of Osteoarthritis and Rheumatoid Arthritis Patients. Int. J. Mol. Sci..

[19] Thiele G.M., Duryee M.J., Hunter C.D., England B.R., Fletcher B.S., Daubach E.C., Pospisil T.P., Klassen L.W., Mikuls T.R. (2020). Immunogenic and Inflammatory Responses to Citrullinated Proteins Are Enhanced Following Modification with Malondialdehyde-Acetaldehyde Adducts. Int. Immunopharmacol..

[20] Ndrepepa G., Kastrati A. (2016). Gamma-glutamyl transferase and cardiovascular disease. Ann. Transl. Med..

[21] Ballatori N., Krance S.M., Notenboom S., Shi S., Tieu K., Hammond C.L. (2009). Glutathione dysregulation and the etiology and progression of human diseases. Biol. Chem..

[22] Pasquini S., Contri C., Borea P.A., Vincenzi F., Varani K. (2021). Adenosine and Inflammation: Here, There and Everywhere. Int. J. Mol. Sci..

[23] Brigelius-Flohé R., Flohé L. (2011). Basic Principles and Emerging Concepts in the Redox Control of Transcription Factors. Antioxid. Redox Signal..

[24] León Fernández O.S., Ajamieh H.H., Berlanga J., Menendez S., Viebahn-Hánsler R., Re L., Carmona A.M. (2008). Ozone oxidative preconditioning is mediated by A1 adenosine receptors in a rat model of liver ischemia/ reperfusion. Tansplantation.

[25] Galiè M., Covi V., Tabaracci G., Malatesta M. (2019). The Role of Nrf2 in the Antioxidant Cellular Response to Medical Ozone Exposure. Int. J. Mol. Sci..

[26] Vaillant J.D., Fraga A., Díaz M.T., Mallok A., Viebahn-Hänsler R., Fahmy Z., Barberá A., Delgado L., Menéndez S., Fernández O.S.L. (2013). Ozone oxidative postconditioning ameliorates joint damage and decreases pro-inflammatory cytokine levels and oxidative stress in PG/PS-induced arthritis in rats. Eur. J. Pharmacol..

[27] Hao W.T., Huang L., Pan W., Le Ren Y. (2022). Antioxidant glutathione inhibits inflammation in synovial fibroblasts via PTEN/PI3K/AKT pathway: An in vitro study. Arch. Rheumatol..

[28] Viebahn-Haensler R., Fernández O.S.L. (2021). Ozone in Medicine. The Low-Dose Ozone Concept and Its Basic Biochemical Mechanisms of Action in Chronic Inflammatory Diseases. Int. J. Mol. Sci..

[29] Oru G.T., Viebhan-Haensler R., Cabreja G.L., Espinosa I.S., Santos B.T., Vega J.C.P., Cintas S.S., Fernández O.S.L. (2017). Medical Ozone Reduces the Risk of γ-Glutamyl Transferase and Alkaline Phosphatase Abnormalities and Oxidative Stress in Rheumatoid Arthritis Patients Treated with Methotrexate. SM J. Arthritis Res..

[30] Fernandez O.S.L., Oru G.T., Vega J.C.P., Fernandez E.G., Viebahn-Hänsler R., Torres-Carballeira R., Cabreja G.L., Mendez R.M. (2020). Ozone + Arthroscopy: Improved Redox Status, Function and Surgical Outcome in Knee Osteoarthritis Patients. Int. J. Innov. Surg..

[31] Zhang H., Liu H., Iles K.E., Liu R.-M., Postlethwait E.M., Laperche Y., Forman H.J. (2006). 4-Hydroxynonenal Induces Rat γ-Glutamyl Transpeptidase through Mitogen-Activated Protein Kinase–Mediated Electrophile Response Element/Nuclear Factor Erythroid 2–Related Factor 2 Signaling. Am. J. Respir. Cell Mol. Biol..

[32] Liao W., Yang Y., Yang H., Qu Y., Song H., Li Q. (2023). Circulating gamma-glutamyl transpeptidase and risk of pancreatic cancer: A prospective cohort study in the UK Biobank. Cancer Med..

[33] Lee Y., Seo J.H. (2023). Potential Causal Association between Elevated Gamma-Glutamyl Transferase Level and Stroke: A Two-Sample Mendelian Randomization Study. Biomolecules.

[34] Kim D., Kim J.H., Lee H., Hong I., Chang Y., Song T.-J. (2023). Association of gamma-glutamyl transferase variability with risk of osteoporotic fractures: A nationwide cohort study. PLoS ONE.

[35] Margis R., Dunand C., Teixeira F.K., Margis-Pinheiro M. (2008). Glutathione peroxidase family—An evolutionary overview. FEBS J..

[36] Kondo N., Kanai T., Okada M. (2023). Rheumatoid Arthritis and Reactive Oxygen Species: A Review. Curr. Issues Mol. Biol..

[37] Mishra R., Singh A., Chandra V., Negi M.P.S., Tripathy B.C., Prakash J., Gupta V. (2012). A comparative analysis of serological parameters and oxidative stress in osteoarthritis and rheumatoid arthritis. Rheumatol. Int..

[38] Peralta C., Xaus C., Bartrons R., Leon O., Gelpi E., Roselló-Catafau J. (2000). Effect of ozone treatment on reactive oxygen species and adenosine production during hepatic ischemia-reperfusion. Free. Radic. Res..

[39] Effendi W.I., Nagano T., Kobayashi K., Nishimura Y. (2020). Focusing on Adenosine Receptors as a Potential Targeted Therapy in Human Diseases. Cells.

[40] Ajamieh H.H., Berlanga J., Merino N., Sanchez G.M., Carmona A.M., Cepero S.M., Giuliani A., Re L., Leon O.S. (2005). Role of protein synthesis in the protection conferred by ozone-oxidative-preconditioning in hepatic ischaemia/reperfusion. Transpl. Int..

[41] Oru G.T., Viebhan-Haensler R., Matos Y.H., Díaz D., Rodríguez M.C.O., Fernández O.S.L. (2018). Medical ozone prevents inflammatory effects from carrageenan-induced knee joint synovitis in rats through A1 adenosine receptor, as well as lipid and protein oxidative damages. J. Sci. Res. Stud..

[42] Mallok A., Vaillant J.D., Soto M.T.D., Viebahn-Hänsler R., Viart M.d.L.A.B., Pérez A.F., Cedeño R.I.D., Fernández O.S.L. (2015). Ozone protective effects against PTZ-induced generalized seizures are mediated by reestablishment of cellular redox balance and A_1_adenosine receptors. Neurol. Res..

[43] Macedo-Júnior S.J., Nascimento F.P., Luiz-Cerutti M., Santos A.R.S. (2021). The role of peripheral adenosine receptors in glutamate-induced pain nociceptive behavior. Purinergic Signal..

[44] Vincenzi F., Pasquini S., Borea P.A., Varani K. (2020). Targeting Adenosine Receptors: A Potential Pharmacological Avenue for Acute and Chronic Pain. Int. J. Mol. Sci..

[45] Declaration of Helsinki, 2013, 7th Revisión. http://jama.md/H1WvEO.

[46] Wichmann B.A., Hill I.D. (1982). Algorithm AS 183: An effcient and portable pseudo-random number generator. Appl. Stat..

[47] McLeod A.I. (1985). Remark AS R58: A Remark on algorithm AS 183. An efficient and portable pseudo-random number generator. J. Appl. Stat..

[48] Aletaha D., Neogi T., Silman A.J., Funovits J., Felson D.T., Bingham C.O., Birnbaum N.S., Burmester G.R., Bykerk V.P., Cohen M.D. (2010). 2010 Rheumatoid arthritis classification criteria: An American College of Rheumatology/European League Against Rheumatism collaborative initiative. Ann. Rheum. Dis..

[49] Cardiel M.H., Abello-Banfi M., Ruiz-Mercado R., Alarcon-Segovia D. (1993). How to measure health status in rheumatoid arthritis in non-English speaking patients: Validation of a Spanish version of the Health Assessment Questionnaire Disability Index (Spanish HAQ-DI). Clin. Exp. Reum..

[50] Merino R.J. (2002). Utilidad diagnóstica de la velocidad de sedimentación globular. Med. Integral.

[51] (1987). Boehringer_Mannheim Biochemica Information. A Revised Biochemical Reference Source. Enzymes for Routine.

[52] Sedlak J., Lindsay R.H. (1968). Estimation of total protein-bound and nonprotein sulfhydryl groups in tissue with Ellman’s reagent. Anal. Biochem..

